# Development and validation of a prognostic classifier based on HIF-1 signaling for hepatocellular carcinoma

**DOI:** 10.18632/aging.102820

**Published:** 2020-02-21

**Authors:** Feiwen Deng, Dong Chen, Xiaoli Wei, Shilin Lu, Xuan Luo, Jincan He, Junting Liu, Tiebao Meng, Anli Yang, Huanwei Chen

**Affiliations:** 1 Department of Liver and Pancreatic Surgery, The Affiliated Foshan Hospital, Sun Yat-Sen University, Foshan 528000, China; 2 Department of Pancreatobiliary Surgery, The First Affiliated Hospital of Sun Yat-Sen University, Guangzhou 510080, China; 3 Department of Medical Oncology, Sun Yat-Sen University Cancer Center, State Key Laboratory of Oncology in South China, Collaborative Innovation Center for Cancer Medicine, Guangzhou 510060, China; 4 Zhongshan School of Medicine, Sun Yat-Sen University, Guangzhou 510008, China; 5 Department of Medical Imaging, Sun Yat-Sen University Cancer Center, State Key Laboratory of Oncology in South China, Collaborative Innovation Center for Cancer Medicine, Guangzhou 510060, China; 6 Department of Breast Oncology, Sun Yat-Sen University Cancer Center, State Key Laboratory of Oncology in South China, Collaborative Innovation Center for Cancer Medicine, Guangzhou 510060, China

**Keywords:** Hypoxia-inducible factor 1, hepatocellular carcinoma, risk score, the cancer genome atlas, gene expression omnibus

## Abstract

HIF-1 (hypoxia-inducible factor 1) signaling played a vital role in HCC (hepatocellular carcinoma) prognosis. We aimed to establish an accurate risk scoring system for HCC prognosis prediction and treatment guidance. 424 samples from TCGA (The Cancer Genome Atlas) and 445 samples from GSE14520 dataset were included as the derivation and validation cohort, respectively. In the derivation cohort, prognostic relevant signatures were selected from sixteen HIF-1 related genes and LASSO regression was adopted for model construction. Tumor-infiltrating immune cells were calculated using CIBERSORT algorithm. HIF-1 signaling significantly increased in HCC samples compared with normal tissues. Scoring system based on SLC2A1, ENO1, LDHA and GAPDH exhibited a continuous predictive ability for OS (overall survival) in HCC patients. PCA and t-SNE analysis confirmed a reliable clustering ability of risk score in both cohorts. Patients were classified into high-risk and low-risk groups and the survival outcomes between the two groups showed significant differences. In the derivation cohort, Cox regression indicated the scoring system was an independent predictor for OS, which was validated in the validation cohort. Different infiltrating immune cells fraction and immune scores were also observed in different groups. Herein, a novel integrated scoring system was developed based on HIF-1 related genes, which would be conducive to the precise treatment of patients.

## INTRODUCTION

Hepatocellular carcinoma (HCC) is the fifth leading cause of cancer-associated death for male worldwide with a high recurrence rate and poor prognosis [1]. Ablative therapies, transarterial chemoembolization (TACE) and surgery have been widely adopted as routine treatments for HCC [2]. Due to the poor clinical prognosis, frequent follow-up and re-treatment are always needed. HCC has been recognized as a heterogeneous disease with various gene expression profiles, biological behavior, and clinical outcomes [3–7].

Although some prognostication systems have been developed for patients with HCC, including hepatoma arterial-embolization prognostic score and assessment for the TACE score, there are limited adopted scoring system reflecting the intratumoral heterogeneous feature of HCC [8, 9]. For cancer patients, the TNM staging system has been recognized as an indicator of clinical outcomes and selection of treatment plan. However, the TNM system only reflects the macroscopic behavior of cancer, including tumor size, lymph node invasion, and distant metastasis, regardless of the intratumoral heterogeneity of patients assigned to the same stage. Besides, the TNM system could not predict individual prognosis for each patient. Hence, a novel scoring system considering intratumoral heterogeneity is in urgent need for prognostic prediction in patients with HCC.

It is well known that O_2_ concentration is significantly decreased in tumor tissues compared with adjacent normal tissues [10]. In the past decades, studies reported that the reduced oxygen induces the activation of hypoxia-inducible factor 1 (HIF-1), which has been recognized as a key factor in reprogramming tumor metabolism, especially in hypoxia response pathways [11–14]. The HIF-1 complex consists of oxygen-regulated HIF-1α and constitutively expressed HIF-1β. HIF-1α plays a key role in the oxygen-sensing pathway and participates in the reprogramming of tumor metabolism. The intratumoral hypoxia-induced HIF-1 leads to an adaptive response to reduce lethal reactive oxygen species (ROS), thereby maintaining cancer survival in a hypoxic environment [15].

Studies have reported the crucial role of HIF-1 in hepatoma epithelial-mesenchymal transition and immune escape [12, 16]. Since traditional TNM stage and clinicopathological factors were unsuitable for prognosis prediction and specific gene profile for individual patients with HCC. A novel scoring system regarding hypoxia-sensing genes may be more reliable for risk stratification of such patients. In the current study, HIF-1 related gene profile was extracted and analyzed for its expression and prognostic value in patients with HCC. A novel oxygen-sensing gene panel was developed using LASSO Cox regression model in the derivation cohort. Furthermore, we verified our panel’s capability of patient stratification and overall survival (OS) prediction in the validation cohort.

## RESULTS

### Expression profile of HIF-1 related genes in HCC

**Figure 1:**
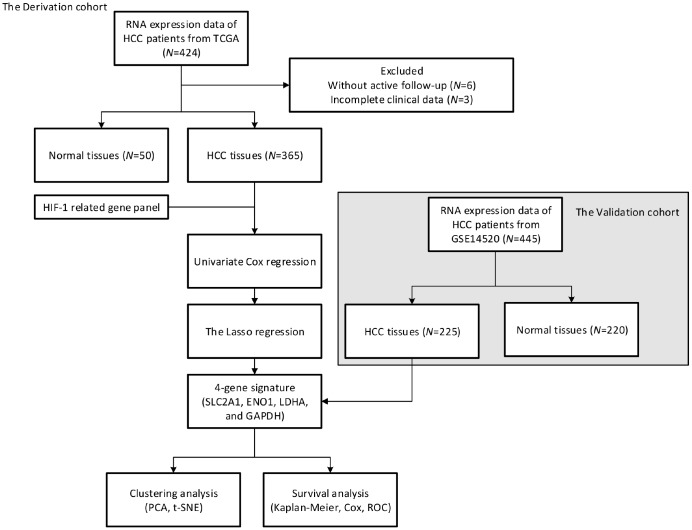
**Study design**. A prognostic classifier was constructed in the derivation cohort (TCGA, N=365) and further validated in the validation cohort (GSE14520, N=225).

**Figure 2:**
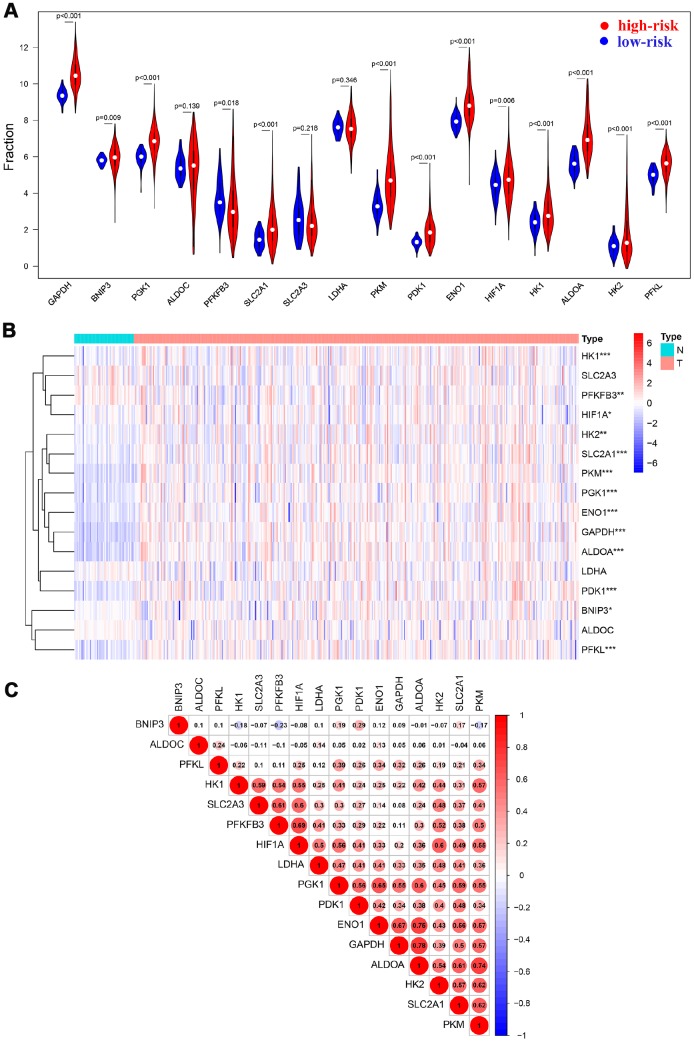
**HIF-1 related genes were significantly upregulated in HCC.** Violin plots (**A**) and heatmap (**B**) showed the expression profile in tumor tissues and normal tissues. Spearman analysis showed a significant correlation among signatures (**C**).

Sixteen HIF-1 related genes were identified in previous study and KEGG pathways, including Solute Carrier Family 2 Member 1 (SLC2A1), Solute Carrier Family 2 Member 3 (SLC2A3), Hexokinase 1 (HK1), Hexokinase 1 (HK2), Aldolase, Fructose-Bisphosphate A (ALDOA), Aldolase, Fructose-Bisphosphate C (ALDOC), Phosphoglycerate Kinase 1 (PGK1), Enolase 1 (ENO1), Pyruvate Kinase M1/2 (PKM), Lactate Dehydrogenase A (LDHA), Pyruvate Dehydrogenase Kinase 1 (PDK1), BCL2 Interacting Protein 3 (BNIP3), Phosphofructokinase, Liver Type (PFKL), Glyceraldehyde-3-Phosphate Dehydrogenase (GAPDH), 6-Phosphofructo-2-Kinase/Fructose-2,6-Biphosphatase 3 (PFKFB3), Hypoxia Inducible Factor 1 Subunit Alpha(HIF1A) [17]. The study design was displayed in Figure 1. 365 patients with HCC in The Cancer Genome Atlas (TCGA) database were enrolled as the derivation cohort. HIF-1 related genes were significantly upregulated in tumors compared with adjacent normal tissues (Figure 2A, 2B). Moreover, correlation analysis validated the association among these genes in HIF-1 related signaling (Figure 2C).

### Prognostic relevance of HIF-1 related genes in HCC

**Figure 3:**
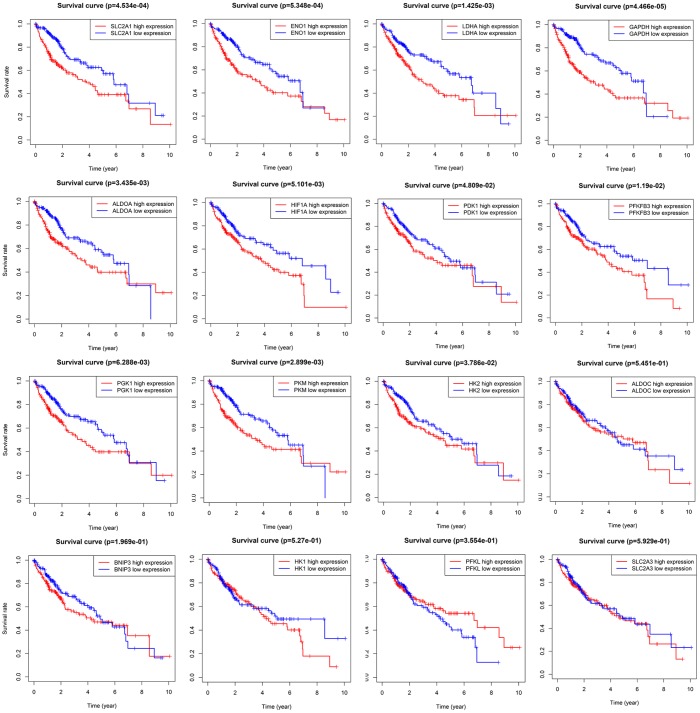
**Survival analysis of sixteen HIF-1 related signatures.** SLC2A1, ENO1, LDHA, ALDOA, GAPDH, HK2, PKM, PGK1, HIF1A, PFKFB3, and PDK1 were prognostic relevant in HCC.

**Figure 4:**
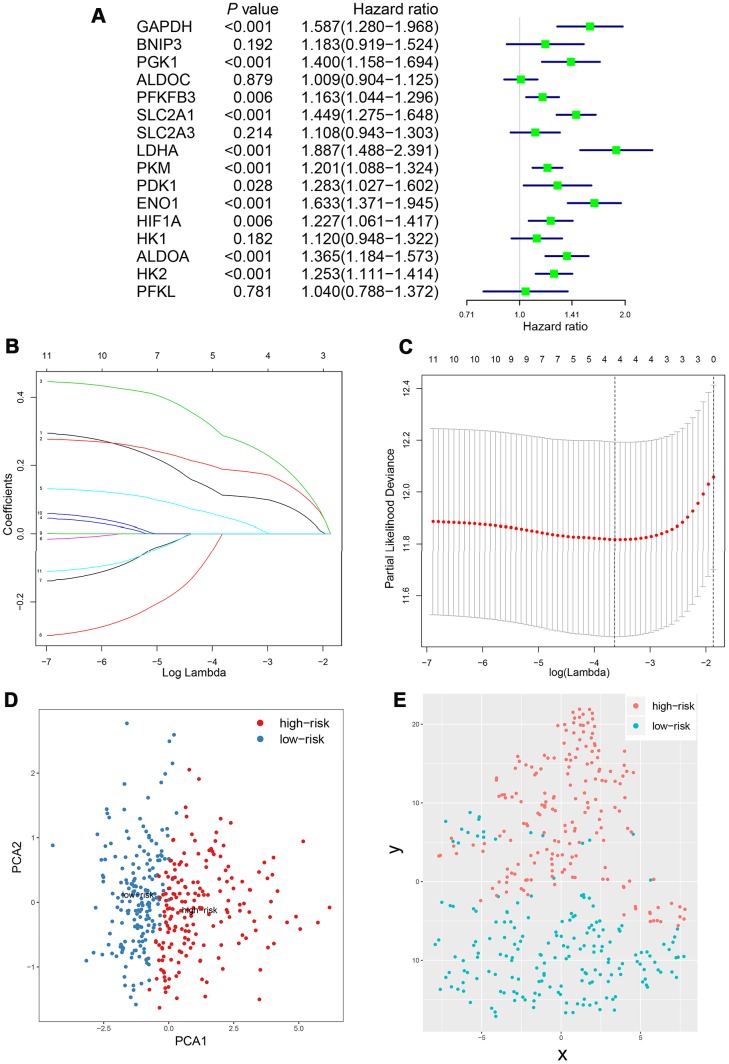
**Construction of integrated risk score based on HIF-1 related genes in the derivation cohort.** SLC2A1, ENO1, LDHA, ALDOA, GAPDH, HK2, PKM, PGK1, HIF1A, PFKFB3, PDK1 were significantly correlated with clinical prognosis in univariate Cox regression model (**A**). The risk score system was constructed using the LASSO Cox regression model (**B**–**C**). PCA and t-SNE analysis revealed an effective clustering ability of four-gene based risk score (**D**–**E**).

Kaplan-Meier analysis and log-rank test were adopted to evaluate the prognostic value of sixteen HIF-1 related genes in HCC (Figure 3). SLC2A1, ENO1, LDHA, ALDOA, GAPDH, HK2, PKM, PGK1, HIF1A, PFKFB3, and PDK1 forecasted poor overall survival (*P*<0.05). The univariate Cox regression model was used to further identify the prognostic relevant genes for overall survival in patients with HCC. Eleven genes, including SLC2A1, ENO1, LDHA, ALDOA, GAPDH, HK2, PKM, PGK1, HIF1A, PFKFB3, PDK1 were significantly correlated with clinical prognosis (P<0.05) (Figure 4A).

### Development of a HIF-1 related gene-based risk model and prognostic score

Furthermore, the identified prognostic features were included in the LASSO Cox regression model for prognostic model and risk score construction (Figure 4B, 4C). Four genes (SLC2A1, ENO1, LDHA, and GAPDH) were selected to establish a risk scoring system due to their integrated prognostic relevance. As SLC2A1, ENO1, LDHA, and GAPDH were recognized as genes which promote anaerobic metabolism in human cells, we further developed an oxygen-sensing specific risk score for patients with HCC. The coefficients for corresponding features were generated according to the partial likelihood deviance and determined with its lowest value at a log λ=-3.45. Four features were included in a formula based on gene expression and the risk score were calculated as follows:

$$\text{risk}\;\text{score}={\text{e}}^{0.111*\text{SLC}2\text{A}1+0.186*\text{ENO}1+0.278*\text{LDHA}+0.042*\text{GAPDH}}$$

The patients were further assigned to the low-risk and high-risk groups by median (risk score=23). PCA and t-SNE analysis confirmed the clustering ability of this four-gene based risk score (Figure 4D, 4E). Functional analysis showed different expression profile between the low-risk and high-risk group (Supplementary Figure 1). GO and KEGG analysis indicated that HIF-1 and hypoxia-related metabolic signaling were enriched in high-risk group (Supplementary Figure 2). Furthermore, PPI network analysis revealed the interactions of these differently expressed genes (Supplementary Figure 3).

### Prognostic value of four-gene based risk score in patients with HCC

**Figure 5:**
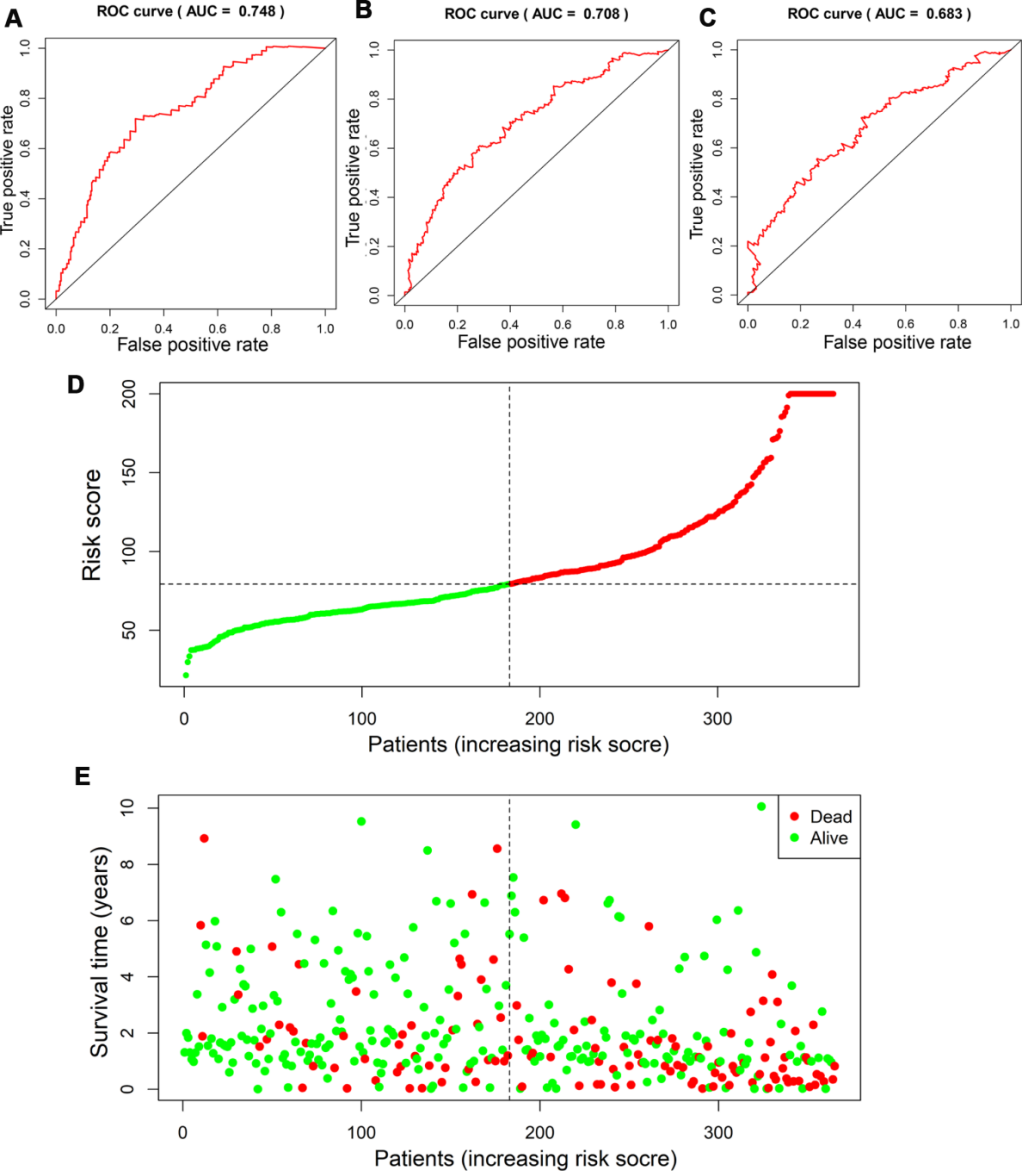
**Assessment of prognostic value of risk score in the derivation cohort.** Time-dependent ROC analysis showed the diagnostic value of risk score at 1 year, 3 years and 5 years after diagnosis (**A**–**C**). Scatter plots showed that different risk scores indicated different survival outcomes in patients with HCC (**D**, **E**).

Based on the scoring system, the prognostic risk of each patient was individualized. The time-dependent receiver operating characteristic (ROC) analysis showed that the area under the curve (AUC) value of four-gene based model was 0.753 (95% CI, 0.673-0.834), 0.710 (95% CI, 0.619-0.800) and 0.669 (95% CI, 0.557-0.781) at 1 year, 3 years and 5 years after diagnosis in the TCGA cohort, respectively (Figure 5A–5C). Moreover, the risk score presented with a better predictive ability compared with the TNM stage during the short-term follow-up (Supplementary Figure 4). The risk score seemed to be more precise in the short-term follow-up of male patients, while more precise in 5-year follow-up of female patients (Supplementary Figure 5). Besides, patients with continuous risk scores harbored various clinical outcomes in different groups (Figure 5D, 5E).

**Figure 6:**
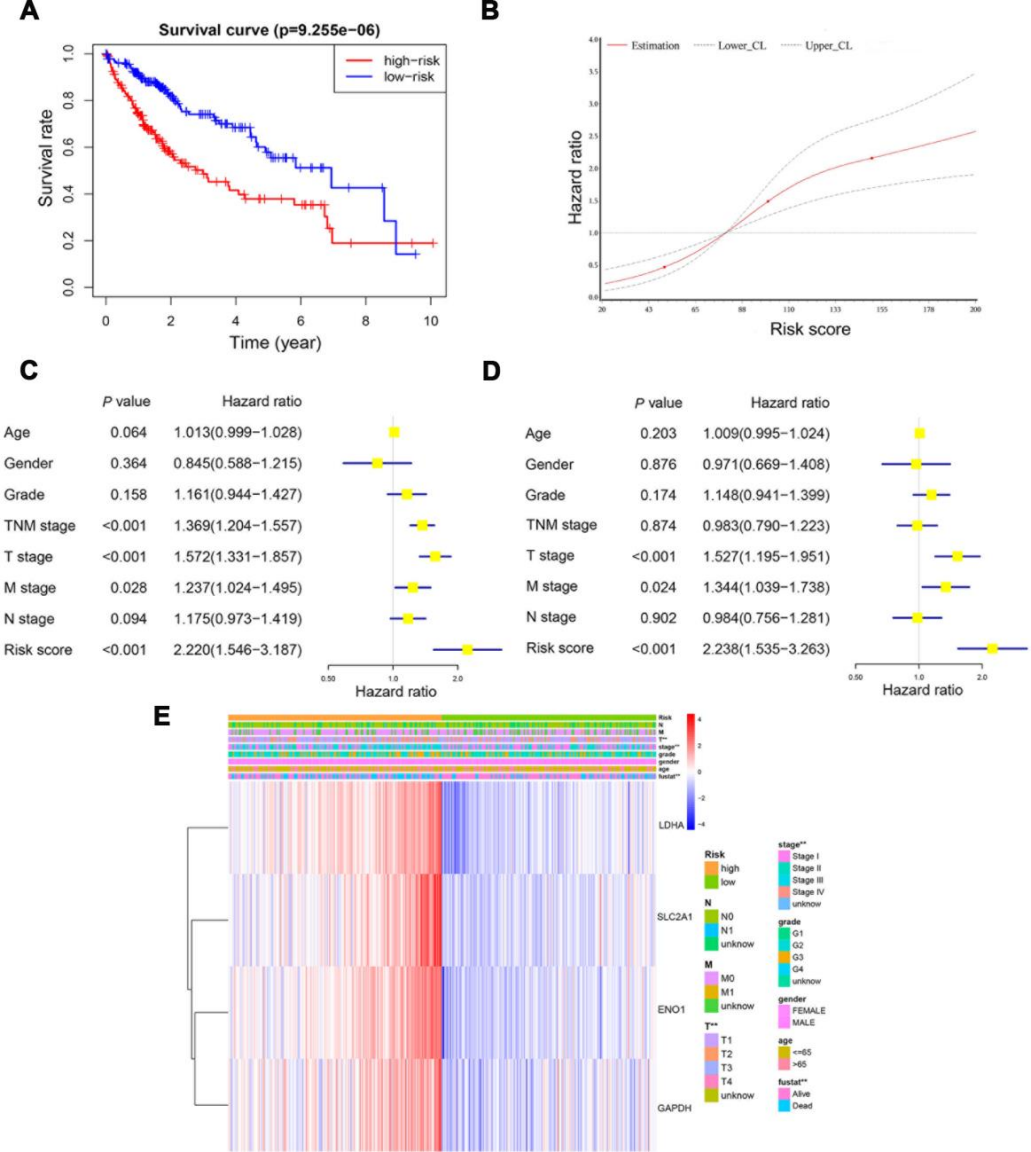
**The risk score was an independent prognostic predictor in the derivation cohort.** Kaplan-Meier plot showed that patients at high-risk exhibited a significantly poorer overall survival compared with those at low-risk (**A**). RCS curve showed an increased risk for overall survival accompanied by corresponding higher risk score (**B**). Univariate and multivariate Cox regression model indicated that the risk score was an independent prognostic predictor for overall survival (**C**, **D**). Chi-square test showed the correlation between risk score and T stage and TNM stage (**E**). *, P<0.05; **, P<0.01.

Kaplan-Meier plot and log-rank test showed that patients at high-risk exhibited a significantly poorer overall survival compared to those at low-risk (Figure 6A). Restricted cubic spline (RCS) revealed that accompanied by the score increases, the risk raised. And the four-gene based risk scoring system was equipped with a continuous predictive ability (Figure 6B). Univariate Cox hazard model in patients with HCC indicated that TNM stage (*HR*: 1.369, 95% *CI*: 1.204−1.557, *P*<0.001), T stage (*HR*: 1.572, 95% *CI*: 1.331−1.857, *P*<0.001), N stage (*HR*: 1.175, 95% CI: 0.973−1.419, P=0.094), M stage (*HR*: 1.237, 95% *CI*: 1.024−1.495, P=0.028) and risk score (HR: 2.220, 95% CI: 1.546−3.187, P<0.001) could predict the overall survival of these patients (Figure 6C). Multivariate Cox hazard model revealed that T stage (HR: 1.527, 95% CI: 1.195−1.951, P<0.001), M stage (HR: 1.344, 95% CI: 1.039−1.738, P=0.024) and the risk score (HR: 2.238, 95% CI: 1.535−3.263, *P*<0.001) could be independent predictors for the above patients (Figure 6D). Furthermore, Chi-square analysis indicated that the risk score was significantly correlated with TNM stage and T stage in patients with HCC (Figure 6E).

### Infiltrating immune cells profile in different groups

**Figure 7:**
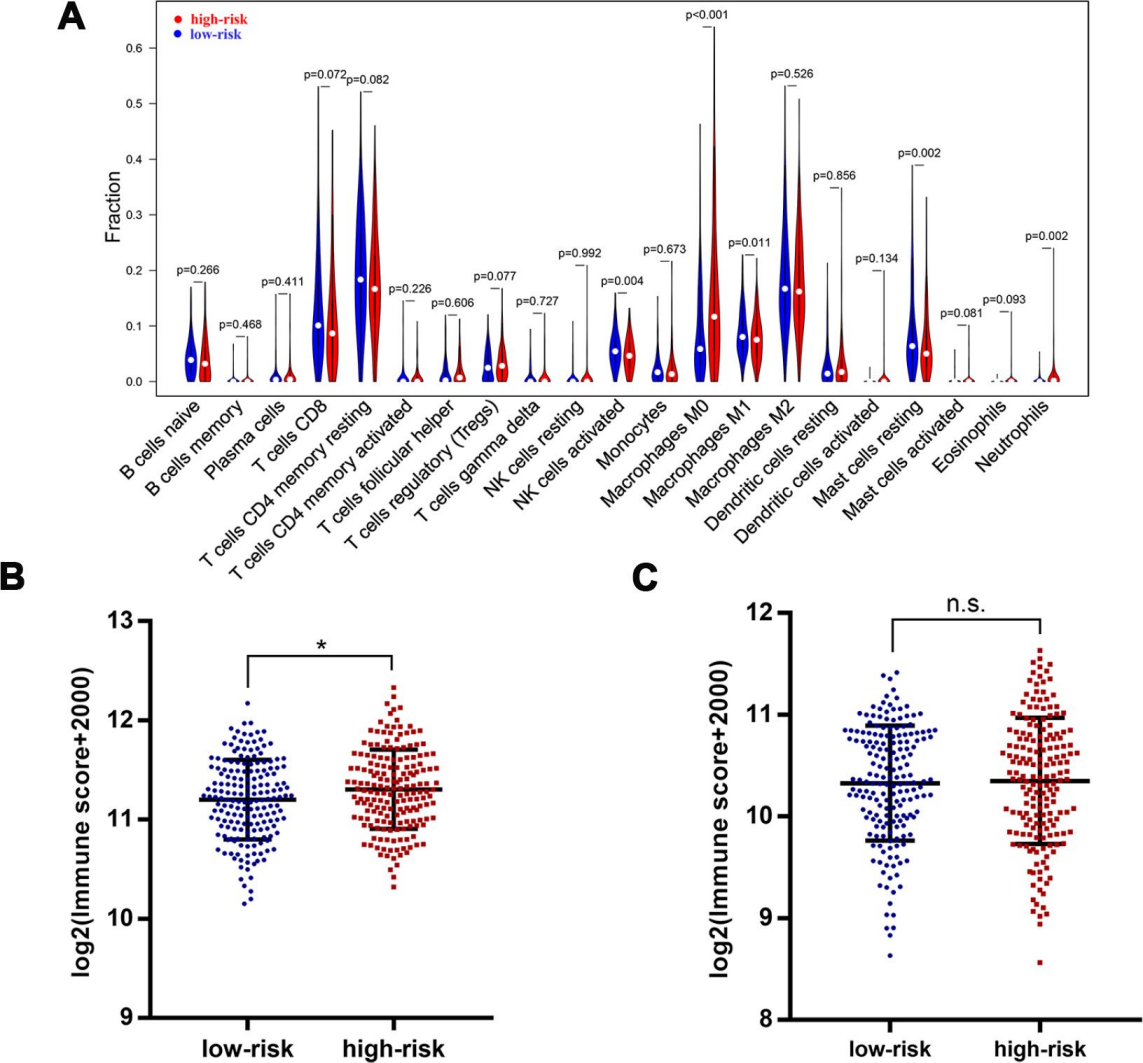
**Immune features in different risk groups.** Violin plot showed tumor-infiltrating immune cells feature in different risk groups (**A**). A significantly higher immune score was observed in patients of high-risk group (**B**), but there was no significant difference for stromal score (**C**). *, P<0.05.

To further identify the difference in tumor-infiltrating immune cells profile between patients with low risk and high risk, we calculated the immune cells fraction using CIBERSORT algorithm (Figure 7A). Patients with high risk exhibited a lower level of CD8+ T cells (*P*=0.072), activated NK cells (*P*=0.004), macrophages M1 (*P*=0.011) and resting mast cells (*P*<0.002). On the other hand, patients with high risk showed a higher level of regulatory T cells (*P*=0.077), macrophages M0 (*P*<0.001) and neutrophils (*P*=0.002). Moreover, an ESTIMATE algorithm was used for tumor microenvironment scoring, by which the immune score and stromal score of HCC were estimated. Patients with high risk showed a significantly higher immune score compared with those at low risk, but there was no significant difference in terms of stromal score (Figure 7B, 7C). These results further confirmed that the four-gene based risk score could serve as a reliable predictor for clinical outcomes and might be an indicator of immune cells infiltration profile in HCC.

### Further validation of the clinical relevance of risk score in GEO dataset

**Figure 8:**
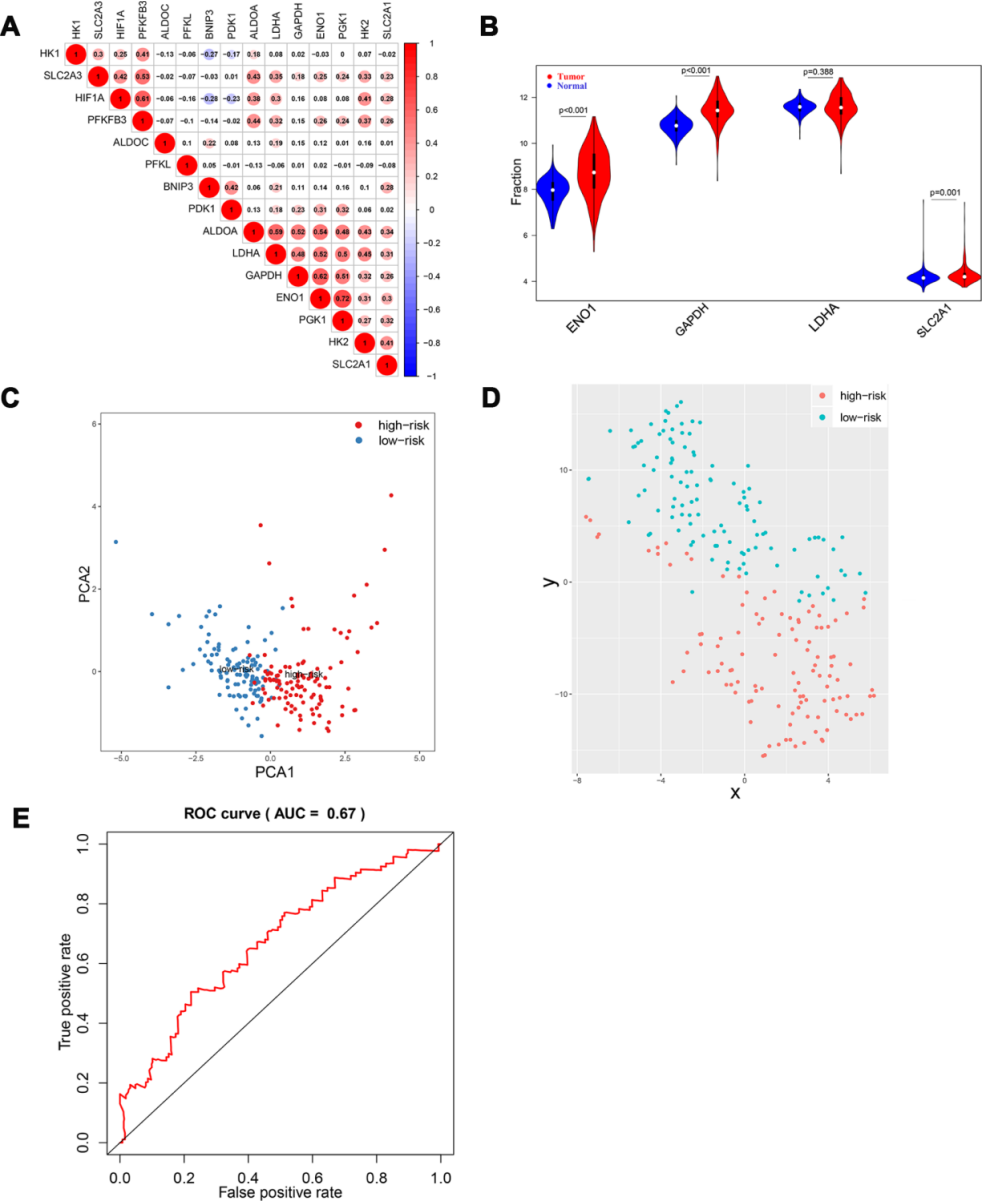
**Validation of HIF-1 related genes and reliability of risk score in the validation cohort.** There were significant correlations between HIF-1 related genes (**A**). Among the four selected genes in the derivation cohort, ENO1, GAPDH, and SLC2A1 were upregulated in HCC (**B**). PCA and t-SNE analysis confirmed the clustering ability of the four-gene panel in the validation cohort (**C**, **D**). Time-dependent ROC analysis showed the diagnostic value of risk score for overall survival at 5 years after diagnosis (**E**).

To further validate the risk score system, a GEO dataset, GSE14520, was included in our study. A total of 445 HCC samples (GPL3921 platform) were analyzed. Results suggested that there was a significant correlation among HIF-1 related genes (Figure 8A). Among the four selected genes, ENO1, GAPDH, and SLC2A1 were significantly upregulated in tumor tissues compared with normal tissues (Figure 8B). The stratification ability of the risk score system was also confirmed by PCA and t-SNE analysis in tumor tissues (N=225) (Figure 8C, 8D). Time-dependent ROC analysis showed an AUC value of 0.671 (95% CI: 0.571-0.772) at 5 years (Figure 8E).

**Figure 9:**
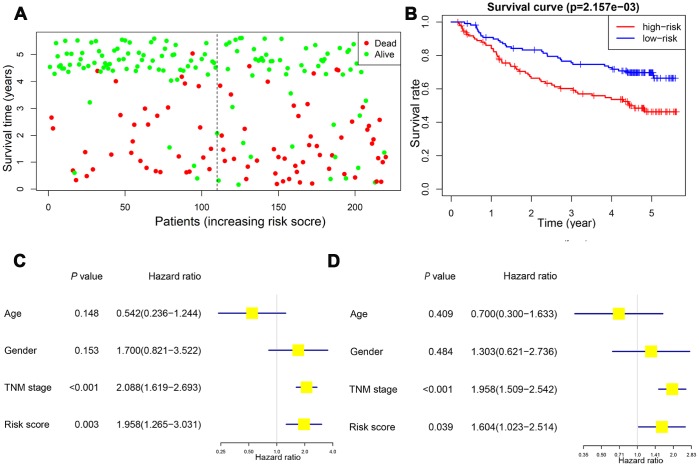
**Validation of the prognostic value of risk score in the validation cohort.** Patients in the high-risk group exhibited a higher incidence of dead events (**A**). Kaplan-Meier plot revealed a poorer overall survival in high-risk group (**B**). Univariate and multivariate Cox regression model confirmed that the risk score was an independent prognostic predictor for overall survival in the validation cohort (**C**, **D**).

Survival analysis was performed to confirm the clinical relevance of the risk score system. Patients in the high-risk group exhibited a higher rate of mortal events (Figure 9A). Kaplan-Meier analysis and log-rank test showed a poorer overall survival in the high-risk group (P<0.01) (Figure 9B). Univariate Cox hazard model indicated that TNM stage (HR: 2.088, 95% CI: 1.619−2.693, P<0.001) and risk score (HR: 1.958, 95% CI: 1.265−3.031, P=0.003) were reliable for survival prediction (Figure 9C). Moreover, the multivariate Cox hazard model confirmed that TNM stage (HR: 1.958, 95% CI: 1.509−2.542, P<0.001) and the risk score (HR: 1.604, 95% CI: 1.023−2.514, P=0.039) were independent prognostic indicators (Figure 9D).

## DISCUSSION

HCC is notorious for poor prognosis and unfavorable response to chemotherapy [18]. Hence, prognosis assessment is crucial for treatment guidance [19, 20]. Traditionally, the AJCC TNM staging system has been considered as a reliable prognostic indicator for patients with HCC. However, the staging is mainly based on the providing macroscopic information and hardly reflects the biological feature and heterogeneity of HCC.

This study serves as the first, to our knowledge, to develop a novel scoring system in regards to HIF-1 related genes. According to previous literature reports, the selected four genes were involved in the hypoxia signaling pathway. The risk score integrating four hypoxia-related signatures was proved to be efficient by survival and ROC analysis. Patients with distinctive survival outcomes and tumor microenvironment were divided into high-risk and low-risk groups by our system, which could help the oncologists select the best therapeutic regimen for specific patients. Previous studies have reported the key role of predictors based on gene expression in tumor prognosis prediction [21–25]. Although HIF-1 signaling has been proved to be upregulated in HCC compared with normal tissues and functioned in metabolism reprogramming, few studies have focused on the integrated predictive value of HIF-1 signals, especially hypoxia-related signatures [26, 27]. Our study aimed to construct a novel integrating risk scoring system using HIF-1 related genes with prognostic relevance. Besides, the large population of TCGA and GEO dataset provided us with sufficient samples and detailed clinical information for model construction and validation, which greatly enhanced the accuracy and reliability of our risk score system.

There are some limitations to our study. Firstly, this integrated scoring system was not further validated by biological experiments. Moreover, the biological mechanism of these genes should be explored in our next studies. Additionally, sixteen HIF-1 related genes were identified and subjected to the LASSO regression model, but only four signatures were finally included in our scoring system. The absence of other signatures might lead to selection bias and should be investigated in future studies. Besides, the integrated risk score should be further validated in multicenter studies before translated into clinical practice. Finally, some therapeutic information, including surgery, ablation, TACE, and targeted therapy, were not included in our study due to the lack of detailed treatment information from both cohorts. Therefore, we were unable to investigate the sensitivity or even the resistance to treatments in high-risk patients.

## CONCLUSIONS

A novel oxygen-sensing related risk score was developed using HIF-1 mediated hypoxia signaling gene panels and validated in two independent cohorts. The individualized risk score could effectively conduct risk stratification, OS prediction, and immune microenvironment judgment for patients with HCC, which would be conducive to the precise treatment of patients.

## MATERIALS AND METHODS

### Study subjects

For TCGA (The Cancer Genome Atlas) and GEO (Gene Expression Omnibus) datasets, the inclusion criteria were as follow: 1) available for OS data and mRNA expression; 2) histologically diagnosed as malignant hepatocellular carcinoma; 3) available for active follow-up data. Patients without detailed or active follow-up were excluded. Due to the unknown identity of patients from the above two datasets, written informed consents were waived by the Ethics Committee of The Affiliated Foshan Hospital, Sun Yat-sen University.

The TCGA dataset was obtained from TCGA using gdc-client. The mRNA expression matrices of GSE14520 were downloaded from the GEO dataset [28]. 424 samples from TCGA and 445 samples from GSE14520 were finally enrolled in our study as the derivation and validation cohort, respectively.

### Development of risk score based on HIF-1 related genes

The data of TCGA was downloaded using gdc-client and the expression data was further normalized using edgeR package in R software (Version 3.5.3). The data of GSE14520 was downloaded from the GEO dataset and the limma package was applied to normalization. Univariate Cox hazard model was adopted to identify prognostic relevant signatures from HIF-1 related genes. Eleven signatures were subjected to the LASSO Cox regression model using the glmnet package [29]. Risk scores were calculated using the generated coefficients and corresponding expression. The patients were further divided into low-risk and high-risk groups according to risk scores.

### Assessment of risk score system

To further confirm the relevance of the aforementioned system, the clustering ability of risk scores was further examined by PCA and t-SNE analysis. Time-dependent ROC analysis was performed and AUC at different time points were calculated to assess the diagnostic value of risk scores [30]. Survival analyses, including Kaplan-Meier plots, log-rank test, univariate and multivariate Cox hazard model were also adopted. GO and KEGG analyses were conducted using clusterProfiler package in R [31]. GO and PPI networks were constructed by Metascape [32].

### Evaluation of infiltrating immune cells, immune score and stromal score

The tumor-infiltrating immune cells fraction was evaluated by uploading the expression matrices to the CIBERSORT, which calculated the fractions according to the LM22 signature with 1000 permutations [33]. To further quantify the tumor microenvironment of patients with HCC, the immune score and stromal score were calculated using the estimate package in R software (Version 3.5.3) [34].

### Statistical analysis

The Chi-square test and the Mann-Whitney U test were used to compare the difference between subgroups. Student's t-test was performed to compare the expression level in different groups when appropriate. Spearman analysis was used for evaluating the correlation between gene expressions. Kaplan-Meier method and Cox regression model were conducted for survival analysis. The Lasso Cox regression model was used to determine the key relevant prognostic phenotypes and the corresponding ideal coefficient. The partial likelihood deviance was calculated by the glmnet package in R software for ideal coefficient selection. Statistical analysis was performed on SAS 9.4 (SAS Institute, Inc), Graphpad Prism 7 (Graphpad) and R 3.5.3 software (https://www.r-project.org). A *P* value <0.05 was determined as statistically significant and all *P* values were two-tailed.

## Supplementary Material

Supplementary Figures
