# Supplementary material for: Development and validation of a prognostic classifier based on HIF-1 signaling for hepatocellular carcinoma
Source: Aging (Albany NY). 2020 Feb 21;12(4):3431–50. doi: 10.18632/aging.102820 (PMC7066907; doi:10.18632/aging.102820)
Supplement: Supplementary Figures [file aging-12-102820-s001..pdf]

SUPPLEMENTARY MATERIALS

Supplementary Figures

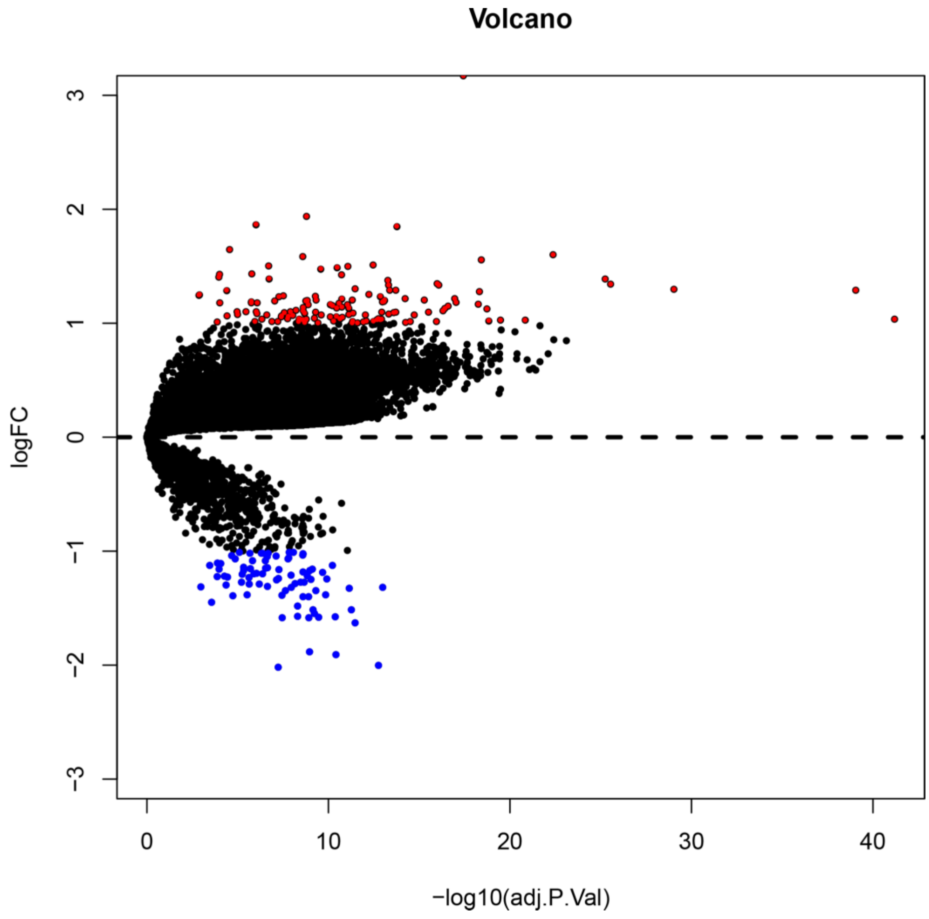

Supplementary Figure 1. The volcano plot showed the difference of expression profile between low-risk and high-risk groups.

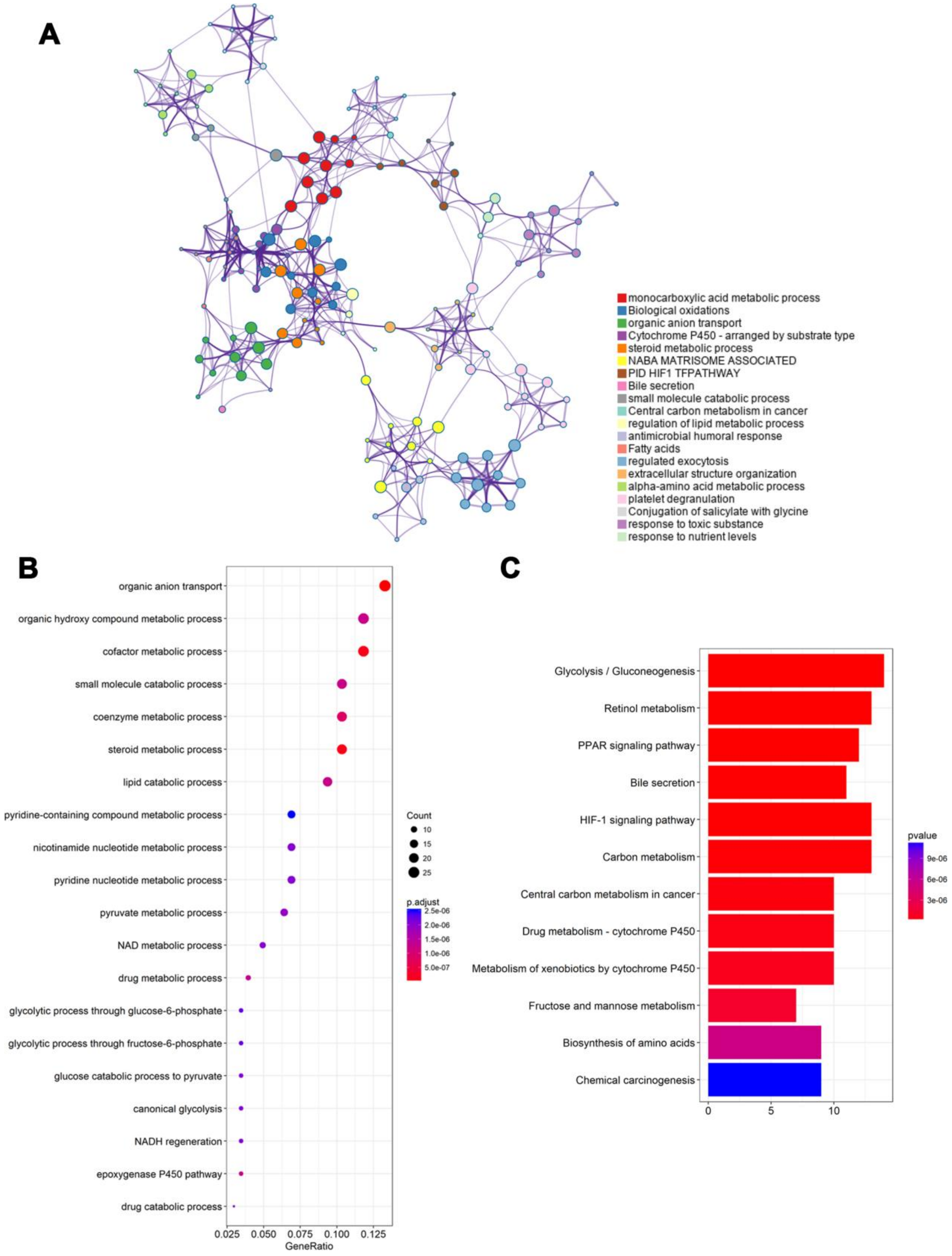

Supplementary Figure 2. The GO and KEGG analysis indicated that HIF-1 signaling pathway was enriched in the high-risk group (**A**–**C**).

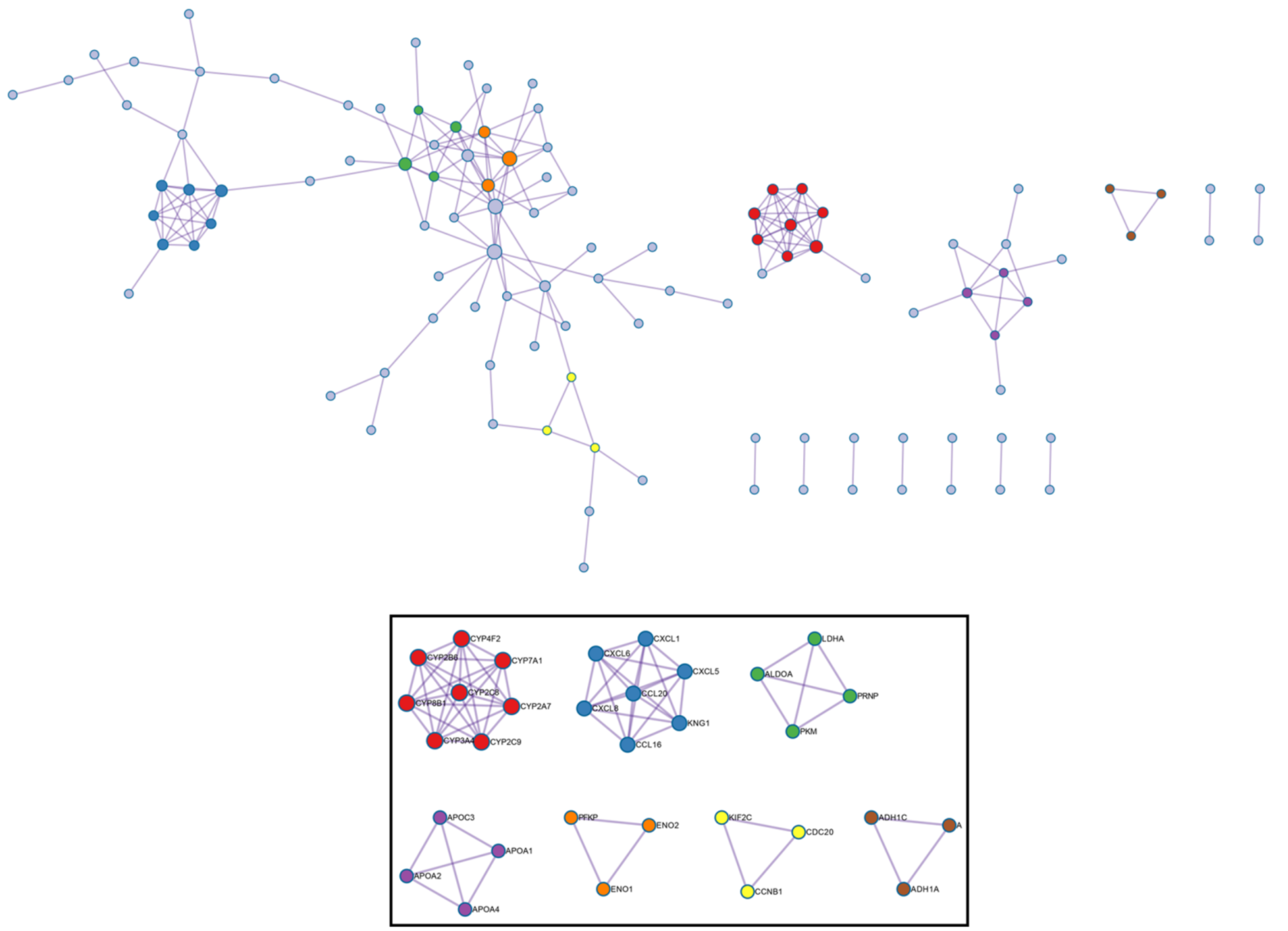

Supplementary Figure 3. PPI network analysis displayed protein interactions of differently expressed genes between low-risk and high-risk groups.

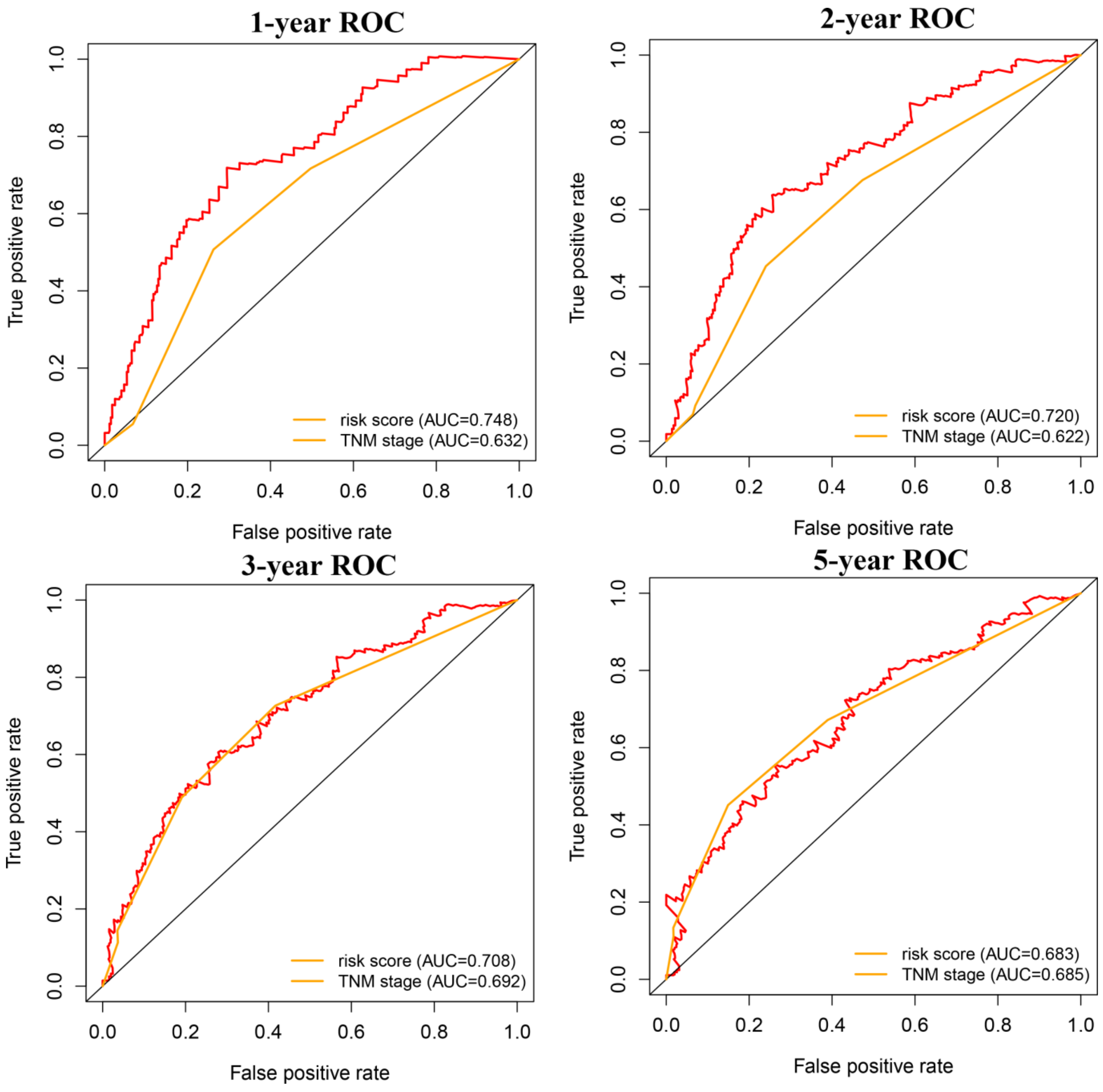

Supplementary Figure 4. The ROC analyses of the risk score and the TNM stage were conducted. The risk score shows better predictive value than the TNM staging in short-term follow-up and still shows the same efficacy as the TNM staging in long-term follow-up.

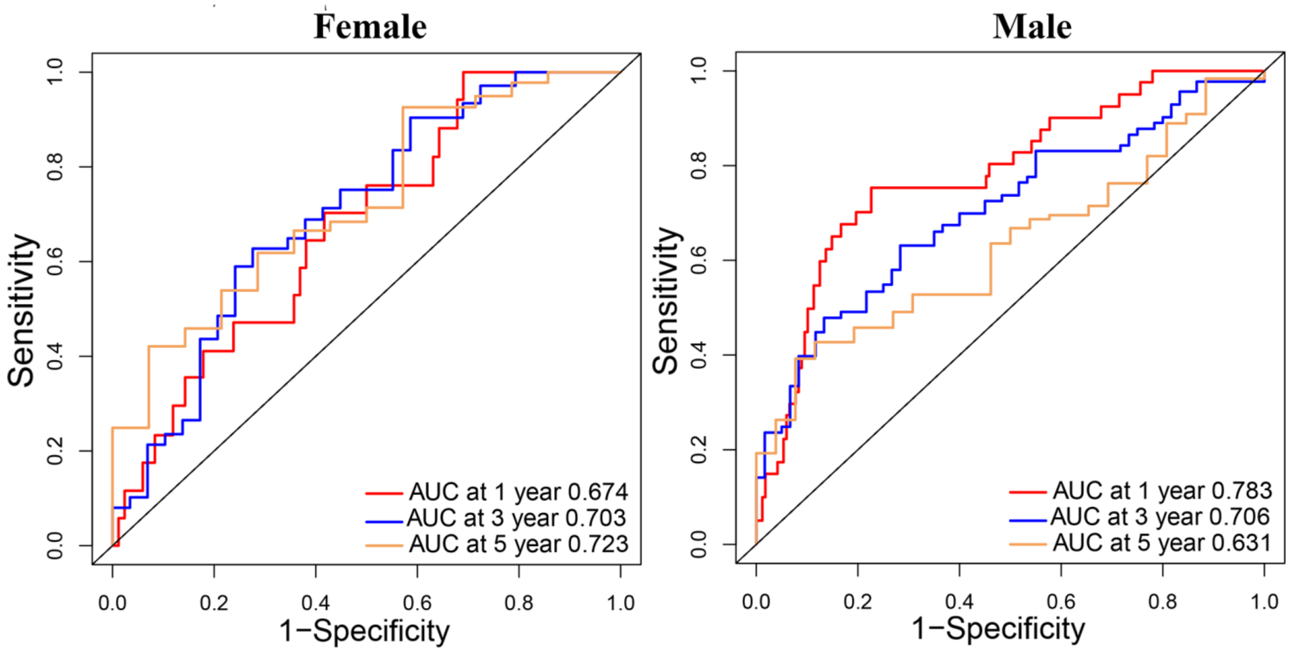

Supplementary Figure 5. The ROC analysis of risk score was performed in male and female patients. The risk score seemed to be more precise in the short-term follow-up of male patients, but be more precise in the long-term follow-up of female patients.
